# Towards a possible aetiology for depressions?

**DOI:** 10.1186/1744-9081-3-47

**Published:** 2007-09-14

**Authors:** Ying Liu, Tore Heiberg, Karl-Ludvig Reichelt

**Affiliations:** 1 Institute of Pediatric Research, Rikshospitalet, N-0027 Oslo, Norway; 2 Oslo Hospital, Ekebergveien 1, N-0192 Oslo, Norway

## Abstract

**Background:**

Since a genetic disposition for depression is probable, there ought to be biochemical changes. Increased peptide levels with relevant bioactivities have been found in urine in a previous investigation, which may be such changes.

**Methods:**

Urine from patients with severe depression according to ICD 10 have been run on reversed phase High Performance Liquid Chromatography, and off line mass spectrometry was performed on some of these peptides.

**Results:**

We find overlapping patterns of peptide peaks in severe depression, but with considerable individuality. Mass spectrometry shows that some of these peptides are probably of dietary origin, because their sequences are found only in certain dietary proteins. Opioids from casein and gliadin are typical examples.

**Conclusion:**

Our data show that the disposition must be polygenetic because some peptide peaks with the same bioactivity are of different length in different patients, but with the same diagnosis. However, some of the peaks are common Peptide increase in urine is found when break down is deficient, and the data presented agree with reports on peptidase deficiencies in depression. Antidepressant drugs decrease the peptide level after about 3 weeks.

## Background

Considerable evidence indicates a genetic disposition for severe depressions [1-4], which of necessity entails chemical changes. The disease takes time to develop, which probably points to unknown substances increasing and, or decreasing until they reach a critical level. We have previously found increased low molecular weight peptides (fragments of proteins) in urine from patients with depression [5,6] diagnosed according to The Diagnostic Manual of Mental Disorder, 3^rd ^edition (DSMIII). A peptide fraction was found that stimulated the uptake of serotonin (5-HT) into platelets [7] Compounds with opioid activity were also found. On account of the confusing and varied patterns and levels of compounds found in urinary profiles from subjects with depression, we wanted to study a severely ill group to try to tease out what is typical. Some of the peptides have been purified guided by serotonin uptake stimulation in platelets [7], opioid receptor binding and/or antibody binding assay [8]. Other peptides have been purified using their mass-spectrometric molecular weight as a guide.

## Patients and methods

Patients were diagnosed mainly by our psychiatrist (TH) according to ICD 10. However, single cases were obtained from various psychiatrists over many years. All 36 patients were severely depressed, needing hospital care and were without medication for at least five weeks. Twentyfour females and 12 males were included in the untreated group with an age range of 26–58 years. Eight were depressed bipolar (F31.5) and we could see no systematic difference in pattern and level of peptides comparing depressed with ICD-10 diagnosis F 32.3, (n = 13) and F 33.3 (n = 15) compared to F 31.5 (n = 8). Therefore all depressions were treated as one group. The treated group used tricyclic anti-depressant or selective serotonin reuptake inhibitors. No difference was found between the two medicated groups and they were therefore treated as one group made up of 18 patients. Twelve females and 6 males with an age range of 23–60. Three males and 8 females were part of the original untreated group, and reanalyzed after 5 weeks of anti-depressive treatment (Table 1). Normal controls were obtained from the hospital staff, nurses, teachers and the Kings Guard regiment. Of these controls none had seen a psychiatrist or psychologist or had suicidal ideas. Their age ranged from 16 to 65 and 118 were female and 99 males. We here report on the urinary state in severe depression only. We have previously found a lack of peptides during mania/hypomania [6].

**Table 1:** Severe Depression and the level of peptides.

Disorder	Depr. Female	Depr. Male	Treated	Control Female	Control Male
Average	812	766	335	252	221
SD	263	257	106	101	69
N	24	13	18	118	99
95% CI					
Lower value	701	611	254	234	208
Higher value	923	921	417	271	235
t:	20.8	17.7			
df:	340	140			
p <	0.001	0.001			

Area is measured in μmeter^2 ^under the 215 nanometer curve integrated by computer. 215 nano-meters was taken to represent the peptide bonds mostly. To control for drugs the UV 280 nm absorption is measured simultaneously. It was difficult to get untreated patients hence the small numbers. P values were obtained using students t test is for females against controls and males against controls. All treated patients (male and female) were on tricyclic anti-depressants or cipramil.

### Urine collection

For pattern analysis the first morning urine and for purification purposes a complete 24 diuresis were collected under supervision (The pattern and levels of compounds were not statistically different comparing morning urine to a 24 h diuresis) and frozen. After thawing, the pH was measured and creatinine determined by the Clinical Chemical Laboratory at Rikshospitalet using standard technique. 0.5 ml urine was pipetted into Costar Spin-x centrifuge filter units (205 Broadway, Cambridge Ma 02139, USA) with cellulose acetate filters of pore radius 0.22 μm and centrifuged at 4000 × g for 30 minutes at 20°C. Filtrate equivalent to 250 nano-moles of creatinine was applied to the column. The column was a C-18 reverse phase column(Vydac C-18 column 0.5 × 25 cm, Hesperia, Ca, USA) detailed elsewhere [9]. Standards obtained from Calbiochem-Novabiochem, AG, Läufelingen, CH-4448, Switzerland and Bachem (Bubendorf, Switzerland) were analyzed after every 11 HPLC runs and spiked urine runs were used when needed.

### Gel filtration

Was performed on Sephadex G-25 columns to separate high and low molecular weight compounds (Dimensions 1.6 × 90 cm run in 0.5 M acetic acid at 0.4 ml/min, application volume 10 ml). After rota-vapor concentration 10 ml of filtered low MW fractions were run on P2 gels (1.6 × 90 cm) again in 0.5 M acetic acid. Off line alkaline hydrolysis and neutralization of 5% aliquots and ninhydrin colouring in an acetate/cyanide buffer was read at 570 nm [8]. This has the advantage that tryptophan is not destroyed by the hydrolysis and the amino acids have equimolar absorption at this wavelength with this method.

### Mass spectrometry

Material obtained from the chromatography peaks was analyzed on the PeSciex API 2000 LC/MS/MS system. The peptides were dissolved in 50% by volume methanol/water/0.01 M formic acid, filtered through Millex GS 0.22 μm filter unit from Millipore, and run in the positive ion mode, and if possible also in the negative ion mode. A blank was run prior to each test sample and because of the high ability for peptides to bind to surfaces, prolonged washing was required. Fragmentation patterns (MS/MS) were compared to that of commercially available standard peptides. The theoretical mass was calculated from tables provided by Micromass UK Ltd, Manchester, UK and compared to the found mass. We chose the average mass different from the mono-isotopic mass. This is the isotopic mix usually found in nature today. To calculate the MW the MW for each amino acid with one Hydrogen removed and an OH group removed is added with addition of one H for the N terminal amino acid and either water (17 daltons) or 16 daltons for C terminal amide and +1 for the positive charge N terminally of NH3 +. The reason for this is that one molecule of water is lost for each peptide bond formed.

### Platelet preparation

Platelets were obtained from healthy post-puberty males and were prepared as described [7]. Because the Fura-2 calcium marker leaks out of platelets that were cooled to 4°C during the preparation, the platelets were prepared with glucose and pyruvate to ensure viability at 20°C [7]. For buffers and details see Pedersen et al 1999 [7]. Platelet count was adjusted to 1.5 × 10^8 ^platelets per ml with buffer.

### Serotonin uptake into platelets

The re-suspended platelets were divided in aliquots of 450 μl stored at 4°C [7] and when used pre-incubated for 10 min at 37°C. For some unknown reason far more stable uptake data were obtained if stored at 4 degrees [10]. The aliquots were incubated with various concentrations of the peptides in 25 μl buffer and for 4 min, and after 2 min (^14^C)-5-HT (Amersham Life Science, UK) in 25 μl was added to a final concentration of 1 μM and 41530 cpm and uptake run for 2 min, the time when the uptake is still linear. For details see Pedersen et al 1999 [7].

This factor and other peptides were purified as described [7,8]. Briefly: a complete 24 h diuresis was reduced by evaporation under reduced pressure (rota-vapor) to 10 ml and applied to gel filtration on G-25 to separate protein from low MW compounds. Peptides were separated from amino acids and salts as described [11] and subsequently the low MW fractions were filtered on P2 gels in 0.5 M acetic acid. Composition of purified peptides was found by hydrolysis in 6 M HCl (Merck) with traces of phenol to protect tyrosine and phenylalanine, in closed glass ampoules at 110°C. for 14 hours. HCL was removed over KOH and P_2_O_5 _in vacuum. Amino acid analysis was performed on the Alpha plus amino acid analyzer (Pharmacia, Uppsala, Sweden) using the ninhydrin technique as ordained for the apparatus. The average from three analyses is given under each peptide. Peptide separation form amino acids is as described [11].

## Results

**Figure 1:**
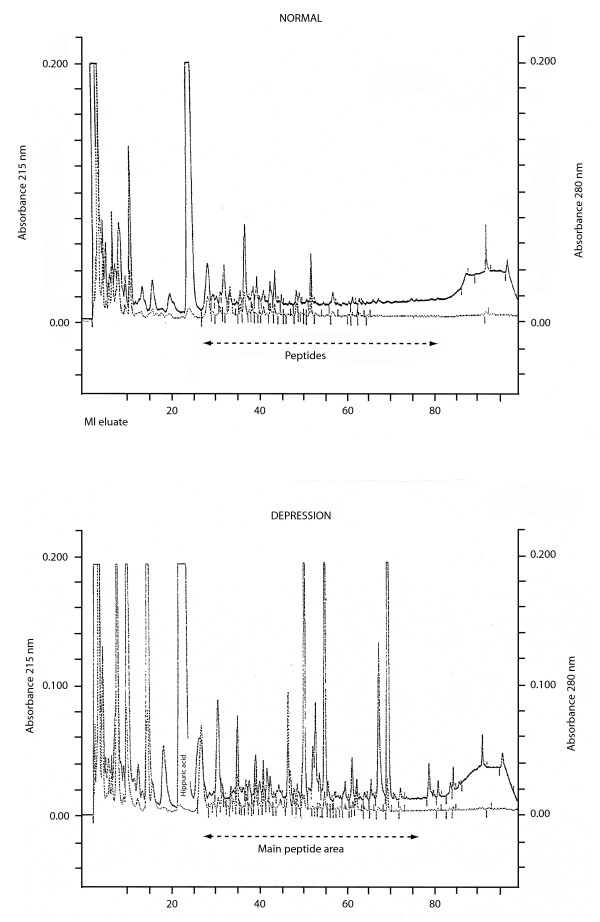
The top trace is a typical 215 nm normal elution pattern from a control on c-18 reverse phase column. (Vydac C-18 protein and peptide column, Vydac, Hesperia, Ca 250 × 4.5 mm) at 30 degrees C [11]. First morning urine equivalent to 250 nano-moles of creatinine was applied. The peaks after hippuric acid were integrated by the HPLC attached computer. The bottom trace is from a severely depressed female patient age 33 yr (Diagnosis: F: 33.3).

**Figure 2:**
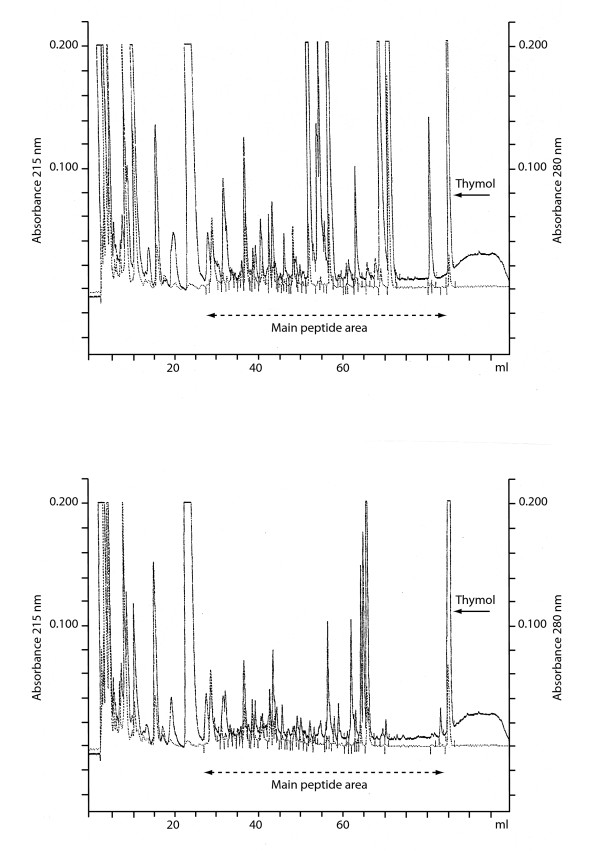
**2a and 2b **HPLC chromatogram from two depressed and hospitalized males with the same diagnosis (ICD 10: F33.3), and age (35 yr). Note the difference in pattern in spite of similar diagnosis, sex and age. The reproducibility of the points of elution running controls after each buffer renewal: For beta-casomorphine (bovine) 1–7: X ± SD = 62.18 ± 0.24 ml (n = 38); 95% Confidence interval = 62.10–62.26. For the beta-casomorphine like peak 1–5: 50.14 ± 0.19 (n = 38) and 95% Confidence interval = 50.08–50.20.

Figure 1 shows a typical HPLC peptidogram from the urine of an asymptomatic control and 1b below is from a psychotic, depressive female age 32(F 33.3). The urinary peptide pattern of two depressed persons (Figures 2a and 2b) both males with ICD 10 diagnosis F 33.3 or serious recurring depressive episode with psychotic symptoms. They both had pronounced symptoms like depressed mood, feeling of guilt, loss of interest and work ability, psychic anxiety and depersonalization. Considerable differences were found in patients with the same sex, age, disorder and degree of depression. For statistical analysis of the peaks eluting after hippuric acid and prior to thymol, the peaks were expressed as area under the 215 nano-meter curve (Table 1). The peptide nature of the compounds has been shown by hydrolysis and re-chromatography of the released amino acids on an amino acid analyzer [8] In Table 1 the quantitative results of untreated, treated and controls is presented. The difference is statistically very significant (Table 1), where male and female controls and treated patients had a significant lower level of peptide excretion (p < 0.05)

**Figure 3:**
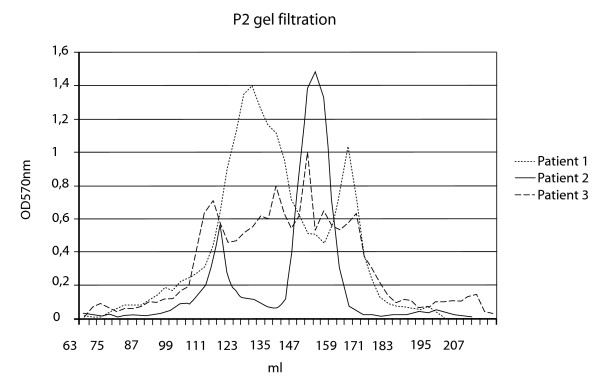
Gel filtration on P 2 gels 1.6 × 90 cm in 0.5 M acetic acid of 24 h complete urine of three patients suffering from depression. (ICD F33.3). The patients were on usual food supply but hospitalized. Whatman 3 MM paper filters was used to filter urine. Elution was monitored by measuring 5% aliquots by off line alkaline hydrolysis and ninhydrin colour developed as described [9], and with an equimolar absorption coefficient for peptides measured at 570 nm for each amino acid.

After gel filtration of urines the most commonly seen pattern (n = 15) of three patients with diagnosis F 33.3 is presented in Figure 3 The gel-filtration constants and the frequency of their occurrence in depression are shown in Table 2. The table again illustrates the chemical heterogeneity of these disorders. We could not classify depressions according to profiles.

**Table 2:** Gel filtration characteristics of low MW peaks separated by size.

Kav	Frequency
0.20	3/27
0.46	11/27
0.50	25/27
0.61	26/27
0.66	20/27
0.74	8/27
0.83	1/27

The frequency of the low MW peak maxima typical for depressions analyzed by gel Filtration expressed as Kav values (A gel-filtration constant) is shown.

Kav = Elution volume – Void volume/Total volume – Void volume. Depression shown here is of psychomotorically retarded psychotic depression. All were drug free and collected over 18 years.

The great variation in peptide chain lengths with the same bio-activity from one patient to the next, makes it important to measure total hydrolysis releasable amino acids. This also increases the sensitivity of the assay because splitting peptide bonds increase the amino groups that can be coloured with ninhydrin [8].

### Serotonin uptake into platelets

**Figure 4:**
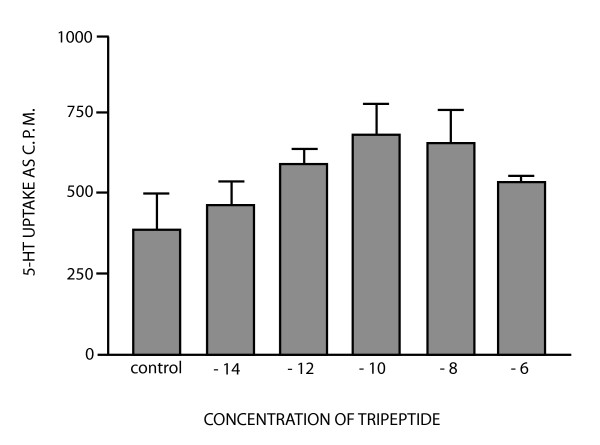
Serotonin uptake stimulation of the pure peptide. Concentration of peptide along the abcissa. Platelets were prepared as outlined in methods. -14 is 10 to the power of -14 M(10^-14 ^M). The typical hormetic dose response curve is shown.

**Figure 5:**
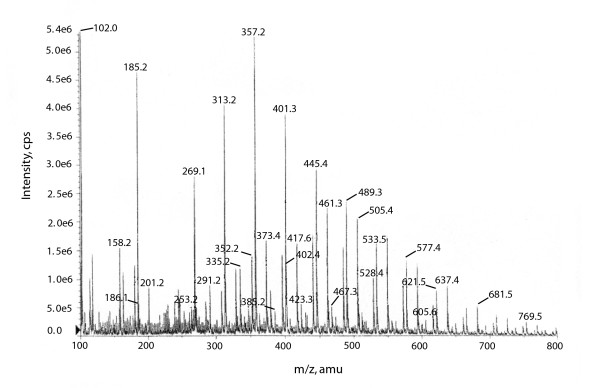
Mass spectrometry of the peak from gel filtration with Kav = 0.61 characteristically partially overlapping with the peak with Kav = 0.66. The serotonin uptake stimulator is seen with mass +1 = 373.4 and a peptide with unknown function and mass +1 of 505.4(Y-P-E-P) which fits deamidated gliadinomoprhine tetrapeptide. Also casomorphine 3–6 with mass +1 = 417.6 is seen (F-P-G-P). A peptide with MW+1 = 489.3 (Expected = 489.5) that may be derived from kappa-casein has a probable structure of L-P-Y-P (casein κ 56–59). Several other peptides will be published separately when elucidated. The figure illustrates the heterogeneity of the gel filtration peaks and the amount of work needed to characterize the many eluted compounds.

In fig 4 the uptake of serotonin into platelets is shown for the final purification step of a tri-peptide with MW = 372.4(expected weight = 372.36) with the M+1 species 373.4 (Figure 5). The probable amino acid E = 1 and G = 1.3 and W (tryptophan) was calculated form UV absorption at 280 nm comparing to synthetic Pyroglu-TrpGlyNH2(pE-W-GNH2)[7]. The uptake curve is typically bell shaped and is found for many peptides [12], and the optimal dose is different from the control with p = 0.001 (n = 9), two tailed. We therefore propose that this peptide may be responsible for serotonin removal into platelets and synapses as found for the amidated tri-peptide [7]. Peptides seen in fig 5 are discussed below, while compounds found in different gel filtration peaks in some, but not all patients are seen in Table 3. Spiking the samples with standards and co-chromatography on HPLC as well as correct amino acid composition was a prerequisite for inclusion in the table. Examples of mass spectrum of the compounds found in the Kav = 0.61 and 0.66 peaks after gel filtration, is given below. The MW is average of three and the following peptides can all be seen in Figure 5:

**Table 3:** Probable structure of some mass spectrometric peaks not shown in fig 5.

Kav of peak	Mass Found	Mass Expected	Structure	HPLC Co-chromatography with
0.61	579.6	579.6	Y-P-F-P-G-NH2	beta-casomorphineb 1–5 amide
0.66	522.3	522.3	Y-P-F-P	beta-casomorphineb 1–4
0.50	888.05	888.05	Y-P-F-P-G-P-I-P	beta-casomorphineb 1–8*
0.50	790.9	790.9	Y-P-F-P-G-P-I	beta-casomorphineb 1–7

b stands for bovine. * Imuno assay for casomorphine 1–8 with antibodies provided by Professor Dr Teschemacher, Giessen, Germany in 4 patients with this HPLC peak came to 120 fentomoles ± 30 (n = 4) while control values from the same peak area in 4 normal controls came to 15 ± 6 (n = 4).

1 From the composite peak with Kav = 0.61 a compound with MW+1 = 505.4 (fig 5) yielded on amino acids Y = 0.8; P = 1.9; traces of G and E = 1. Found mass+1 = 505.4 (Expected mass +1 = 505.54). This fits gliadin-morphine 1–4 deamidated or Y-P-E-P.

2 Also in the Kav = 0.66 and 0.61 peak beta-casomorphine 3–6 with amino acid composition F = 0.9; P = 2; G = 1.4. (Glycine always appear higher than expected even in standard synthetic compounds). Found MW +1 = 417.5(Expected = 417.4), which thus agrees with F-P-G-P. (Figure 5, the MW came out as 417.6) or casomorphine 3–6.

3 MW+1 = 357.2 from the Kav = 0.66 peak likewise fits Lactoferrin 310–313 or SP-P-G or theoretical MW+1 = 357.37 and hydrolysis gave P(2), S(0,9) and G (1.4).

4 With a MW+1 of MW+1 = 489.3 (Expected = 489.5) probably from kappa-casein has a reasonable structure of L-P-Y-P (casein kappa 56–59). Amino acid composition was quite varying but V = 1, Y = 0.6, P = 2. (Glycine was sometimes found but varied from 0 to 1.)

We also obtained a peak that eluted with substance P (co-chromatography after spiking). However, it clogged (for unknown reason) the capillary electrode in the mass spectrometer, so we were unable to obtain its mass and fragmentation pattern.

## Discussion

We have found substantial increase in peptide excretion in severe depression. To our surprise the level was found to be decreased in treated patients. Increase in peptides especially in urine is usually due to peptidase deficiency or inhibition [13,14]. The presented data indicate that severely depressed patients show hyper-peptiduria as would be expected if peptidase defects were present. Peptidase deficiencies have been found in depression and melancholic states [15-19]. Because the level and pattern of peptides differ, and differing chain lengths of peptides with the same activities are found, this points to a heterogeneity of genetics. Different families may have a different sets of dysfunctional enzymes probably peptidases or peptidase binding proteins. This may explain the difficulty of pinpointing a specific gene as the genetic disposition in depression. We suggest that we have depression causing genes which may cause formation of different but overlapping peptidases or peptidase regulating proteins, and that the mediators of depression may be peptides regulating the uptake and release of different transmitters. Since antidepressant medication reduced the level of peptides (table 1) this could be due to peptidase induction as has been shown for neuroleptic medication. It remains to be seen if peptide level correlates with the degree of depression. The presence of opioids may explain the psychotic features of our patients because opioids have been shown to cause increased dopamine in the synaptic cleft by inhibiting reuptake [20] An exogenous supply of peptides may also explain the often seen and peculiar fluctuating course of the disorder with morning worsening and afternoon a relative high or agitation. Increased reuptake of serotonin into platelets may have relevance to the reported changes in serotonin transport into platelets in depression [21,22]. Both the tri-cyclic antidepressants and selective serotonin re-uptake inhibitors have the opposite effect on serotonin uptake. We have previously found the tri-peptide but amidated, in autistic patients' urine and this tri-peptide stimulated the serotonin transporter mediated uptake of serotonin in hamster ovarial cells transfected with the human serotonin transporter gene [23]. That tri-peptide doubled the serotonin content of platelets when injected into pups subcutaneously [24]. 5 HT 2a receptors are increased in brain tissue and platelets in depression [25] which would agree with decreased levels in the synaptic cleft due to stimulated uptake. Also the decreased 5HIAA (5-hydroxy indole-acetic acid) in suicidal patients fit an increased uptake [26]. The tri-peptide sequence has only been found in reelin, which is a matrix proteinase [27]. That food derived peptides are taken up has been demonstrated [28,29] and is increased by peptidase defects [30]. Depression has been found in cases of coeliac disease [31,32] indicating that such a mechanism is not unreasonable. Considerable differences in rates in different cultures may also thus be explainable. Other groups have found increases in some peptides in depression. Thus TRH (pE-H-P-NH2) has been found increased in CSF [33,34]; beta-endorphin [35] and an unspecified opioid fraction 1 measured by receptor binding [36,37]. Substance P was also found increased in CSF [38], and delta sleep factor increased in plasma [39,40]. Also plasma arginine vasopressin increase in depression was inversely related to daytime motor activity [41]. Furthermore post partum psychosis may be mediated by different human caso-morphines [42] and shows depressive traits. Since peptides in general are excellent peptidase inhibitors [43] and peptides also have strong tendencies to form complexes [44,45] and bind to other molecules and membranes [46,47], a rather complex situation with varied results can be envisioned. Bell shaped dose responses are common to many peptides and add to this complicated picture [12]. The nature of some of these peptides is shared with schizophrenia and may constitute the common features of these disorders [48]. Possibly relevant to a gut-brain axis in depression is epidemiological data showing very high frequency of depression in irritable bowel syndrome [49-51]. Future work is needed to elucidate if there is a correlation of peptide increase and degree of depression and to look for any correlations between individual peptide peaks and specific symptoms of this disorder.

## Conclusion

In severe cases of hospitalized patients with depression, we find peptide increase in urine. After treatment the level is decreased. Some of these peptides affect serotonin uptake and others show exorphine like characteristics. Our study is based on a small number of patients due to problems of getting untreated ones. We do not know how general or specific these findings are to the depression spectrum. However, our data are compatible with reported decreased peptidase levels found by others in depression. If correct the profound heterogeneity casts doubt on most double blind treatment trials in depression.

## Authors' contributions

LY carried out the serotonin uptake studies, TH the diagnosis of patients and urine collection, KLR did the urine analysis, mass spectrometry and administration of the project.
